# Interrogating intervention delivery and participants’ emotional states to improve engagement and implementation: A realist informed multiple case study evaluation of Engager

**DOI:** 10.1371/journal.pone.0270691

**Published:** 2022-07-14

**Authors:** Lauren Weston, Sarah Rybczynska-Bunt, Cath Quinn, Charlotte Lennox, Mike Maguire, Mark Pearson, Alex Stirzaker, Graham Durcan, Caroline Stevenson, Jonathan Graham, Lauren Carroll, Rebecca Greer, Mark Haddad, Rachael Hunter, Rob Anderson, Roxanne Todd, Sara Goodier, Sarah Brand, Susan Michie, Tim Kirkpatrick, Sarah Leonard, Tirril Harris, William Henley, Jenny Shaw, Christabel Owens, Richard Byng

**Affiliations:** 1 Community and Primary Care Research Group, Faculty of Health, University of Plymouth, Plymouth, Devon, United Kingdom; 2 Division of Psychology and Mental Health, Faculty of Biology, Medicine and Health, University of Manchester, Manchester, United Kingdom; 3 Department of Criminology, University of South Wales, Cardiff, United Kingdom; 4 Hull York Medical School, Faculty of Health Sciences, University of Hull, Hull, United Kingdom; 5 South West Mental Health Clinical Network, Taunton, United Kingdom; 6 Centre for Mental Health, London, United Kingdom; 7 Division of Health Services Research and Management, School of Health Sciences, City, University of London, London, United Kingdom; 8 Comprehensive Clinical Trials Unit, University College London, London, United Kingdom; 9 Medical School, College of Medicine and Health, University of Exeter, Exeter, United Kingdom; 10 Division of Psychology and Language Sciences, Research Department of Clinical, Educational and Health Psychology, Kings College London, London, United Kingdom; Flinders University, AUSTRALIA

## Abstract

**Background:**

‘Engager’ is an innovative ‘through-the-gate’ complex care intervention for male prison-leavers with common mental health problems. In parallel to the randomised-controlled trial of Engager (Trial registration number: ISRCTN11707331), a set of process evaluation analyses were undertaken. This paper reports on the depth multiple case study analysis part of the process evaluation, exploring how a sub-sample of prison-leavers engaged and responded to the intervention offer of one-to-one support during their re-integration into the community.

**Methods:**

To understand intervention delivery and what response it elicited in individuals, we used a realist-informed qualitative multiple ‘case’ studies approach. We scrutinised how intervention component delivery lead to outcomes by examining underlying causal pathways or ‘mechanisms’ that promoted or hindered progress towards personal outcomes. ‘Cases’ (n = 24) were prison-leavers from the intervention arm of the trial. We collected practitioner activity logs and conducted semi-structured interviews with prison-leavers and Engager/other service practitioners. We mapped data for each case against the intervention logic model and then used Bhaskar’s (2016) ‘DREIC’ analytic process to categorise cases according to extent of intervention delivery, outcomes evidenced, and contributing factors behind engagement or disengagement and progress achieved.

**Results:**

There were variations in the dose and session focus of the intervention delivery, and how different participants responded. Participants sustaining long-term engagement and sustained change reached a state of ‘crises but coping’. We found evidence that several components of the intervention were key to achieving this: trusting relationships, therapeutic work delivered well and over time; and an in-depth shared understanding of needs, concerns, and goals between the practitioner and participants. Those who disengaged were in one of the following states: ‘Crises and chaos’, ‘Resigned acceptance’, ‘Honeymoon’ or ‘Wilful withdrawal’.

**Conclusions:**

We demonstrate that the ‘implementability’ of an intervention can be explained by examining the delivery of core intervention components in relation to the responses elicited in the participants. Core delivery mechanisms often had to be ‘triggered’ numerous times to produce sustained change. The improvements achieved, sustained, and valued by participants were not always reflected in the quantitative measures recorded in the RCT. The compatibility between the practitioner, participant and setting were continually at risk of being undermined by implementation failure as well as changing external circumstances and participants’ own weaknesses.

**Trial registration number:**

**ISRCTN11707331,** Wales Research Ethics Committee, Registered 02-04-2016—Retrospectively registered https://doi.org/10.1186/ISRCTN11707331.

## 1 Background

The success of an intervention depends on how fully and how well it is put into practice, engages the intended population, and generates changes in keeping with its intrinsic logic. Process evaluations provide essential insight into implementation, including the extent to which interventions were delivered as intended (‘fidelity’), variations in engagement, and specific circumstances under which outcomes were attained [1–4]. The knowledge gained identifies where implementation worked well (and may be translated into future practice) [5], and where there were inadequacies in intervention delivery or theory [6, 7]. Here we report a pragmatic and theory driven approach to complex intervention evaluation, providing an exemplar for testing underlying programme theory about how complex interventions work, taking into account the contributions of intervention participants.

### 1.1 The Engager intervention

Engager was developed in response to an omission in mental health focused RCTs carried out in prison settings [8]; this despite the high prevalence of mental health problems reported among prison populations [9–11]. For example, compared to the general population, research has found that prisoners are more likely to display: higher rates of drug dependency and hazardous alcohol use; anxiety and depression; and greater instances of self-harm and suicide ideation [12–14]. There is also substantial comorbidity, particularly in regard to substance use; and co-occurring social problems such as unstable housing or homelessness, familial relationship breakdowns; and unemployment or financial concerns [15, 16]. Despite the prevalence of mental health problems among prison populations, they tend to have minimal access to mental health care, and poor continuity of service provision on release from prison [15, 17, 18]. Similarly, although complex relationships between mental health, substance misuse, social exclusion and criminal behaviour are known to exist, they tend to be studied separately and interventions designed to address them developed in isolation from each other [1]. Therefore Engager was a novel intervention designed to bridge this gap in service provision for offenders between prison and the community.

Engager was a parallel two-group randomised controlled trial with 1:1 individual allocation to either: (a) standard care plus the intervention (intervention group) or (b) standard care alone (control group) across two investigation centres (South West and North West of England). Participants were 280 prisoners (140 per group) meeting eligibility criteria: one or more common mental health problem (anxiety, depression, PTSD), serving a sentence of less than 2 years, and due to be released within four months from the date of recruitment to the RCT.

Participants recruited to the intervention arm of the Engager Randomised Control Trial (RCT) worked one-to-one with a practitioner, who supported their often co-morbid, needs [12–14, 19] as they transitioned from prison back into the community. Engager Practitioners were recruited from a range of health and social care backgrounds, including support workers, substance use workers, and third sector providers; they often had limited therapeutic experience but were trained and supervised by clinical leads. Using a person-centred approach, the intervention was designed to be tailored to individuals’ psychosocial needs, embedding multi-agency working by overcoming service barriers and facilitating access to community services [17–19]. Based on established principles of behaviour change [20, 21] and collaborative care [22, 23], Engager was developed using realist methods [23, 24], and therapeutically underpinned by a mentalisation-based approach (MBA) [25]. MBA involves helping people develop the ability to understand actions by others and one’s self in terms of thoughts, feelings, wishes and desires, learning how to respond more adaptively in moments of distress.

The realist formative process evaluation (FPE) of the pilot trial built, tested, and refined the underlying Engager intervention theory, producing an evidence-based intervention logic model [25]. From the pilot trial FPE data a logic model was produced and was then represented diagrammatically (see S1 File). See [25] for the programme theory development process and detailed theory as intended for delivery in the Engager RCT. This included an implementation delivery platform which sought to facilitate standardisation of intervention delivery within and between sites. The components of the implementation delivery platform included: a comprehensive manual describing actions for Engager practitioners and supervisors to guide their delivery of intervention components; a training programme for supervisors and Engager practitioners, including the logic and rationale of the model and mentalisation-based approaches; a programme of supervision for Engager practitioners to receive support while delivering prison and community care, both one-to-one with supervisors and team supervision incorporating informal peer-to-peer discussions and formal monthly meetings attended by members of the research team to provide meta-supervision; and organisational agreements and support for clinical governance needs (record keeping, line management etc) and inter-organisational working (with prison and probation). Some flexibility in intensity of work and number of contacts was permitted as required by individual participant need; with a minimum ‘dose’ of two pre-release contacts and eight post-release contacts over a one-four month pre- and three-five month post- release period (see [26] for the trial protocol).

### 1.2 The realist-informed process evaluation

Realist evaluation seeks to explain how interventions work ‘for whom, why, and under what circumstances’ [27]. This involves scrutinising how intervention components produce desired outcome patterns by examining mechanisms (underlying causal pathways) that promote or hinder outcome attainment and intervention effectiveness [28]. It is posited that mechanisms (the ways in which people respond to the resource on offer) may only activate in a certain set of circumstances (the ‘context’); and it is a combination of this context and the associated underlying mechanisms that have causal power and lead to intervention outcome attainment. Realist evaluation can contribute to understanding the interacting, constraining and enabling factors that determine how complex interventions take effect [29–31].

Developed using realist informed methods [24, 27], the Engager logic model comprised:

- Core components; the series of actions the Engager Practitioners were asked to deliver;
- Internal responses or ‘mechanisms’ that the components were expected to activate in intervention participants; and
- Tangible ‘outcomes’ that the intervention aspired to produce for prison leavers as a result.

Engager, a novel intervention, was theoretically developed given the lack of previous innovation of mental health interventions for prison-leavers. The exploratory and explanatory depth multiple case study analysis reported in this paper aimed to:

- Determine the degree to which core components were delivered, mechanisms activated, and intended outcomes achieved in line with the theoretical logic model;
- Explore individual participants’ engagement with the intervention over time; and
- Identify aspects of intervention theory/ delivery that could be improved.

This analysis was combined with the other analyses which comprised the parallel process evaluation of the RCT and contributed to the wider interpretation of the both results of the RCT and the refinement of the intervention and its delivery [32].

## 2 Methods

### 2.1 Study design and setting

A realist informed, longitudinal, qualitative, depth, multiple case study design was used. Individual Engager participants in receipt of the intervention were ‘cases’ (the unit of delivery for the intervention). The study setting comprised the two intervention delivery regions (North West and South West) of England.

A trial registration application for the Engager randomised-controlled trial (Trial registration number: ISRCTN11707331) was made in December 2015 through the NIHR portfolio registration system (before recruitment) but due to administrative delays the ISRCTN registration date was 4/02/2016 while recruitment started 14/1/2016. The authors confirm that all ongoing and related trials for this intervention are registered.

### 2.2 Sample

All intervention arm participants (n = 140) were given the opportunity to participate in additional data collection for the process evaluation (PE). We purposively sampled potential PE participants using a framework based on ‘Clinical Outcomes in Routine Evaluation–Outcome Measure’ (CORE-OM) [33] baseline scores (the primary trial outcome measure assessing psychological distress). From this, 24 were opportunity sampled to ensure that we could contact them and collect data within the timeline. Although small sample sizes are often noted in terms of potential limited generalisability of findings; we followed guidance for traditional qualitative research when determining our sample source and size [34]. That is to say, we used a) a relatively sample size in order to support the depth of longitudinal case-oriented analyses fundamental to qualitative research; and b) purposive sampling to ensure cases selected contained a breadth of richly-textured contextual and circumstantial variation, crucial for testing programme theory in realist enquiry.

### 2.3 Data collection

Sources of data comprised face-to-face semi-structured interviews with participants at three time-points where possible (baseline/ pre-release; follow-up at three-six months post-release; and further follow up 12 months post-release); as well as interviews with Engager practitioners, and practitioners from other services (e.g. probation officers, support housing providers). We also collected practitioner activity logs which included daily timesheet activities (e.g. face-to-face sessions with prison-leavers and community liaison work) and detailed session records including purpose, session content, resources used, goals, events and actions. See Table 1 in S2 File for a full list of data sources collected for each case study participant. Interviews were audio-recorded and transcribed verbatim. The realist informed interview schedules [29, 35] were guided by the realist intervention theories developed in the FPE [24], and tailored to data collection time points.

### 2.4 Ethics approval and consent to participate

Ethics approval from the relevant ethics committees were obtained, as reported in the trial protocol. Specifically, we obtained ethical approval from East of England–Essex Research Ethics Committee (reference number 13/EE/0249); National Offender Management Service NRC (reference number 2013–187); National Offender Management Service (ref: 2015–283) approval and local governance approvals for each site (Devon Partnership Trust NHS Trust, Dorset Hospital University Foundation NHS Trust and Lancashire Care NHS Foundation Trust). The study was adopted by the National Institute for Health Research (NIHR) Clinical Research Network and the study sponsor is Devon Partnership NHS Trust. All prison-leavers participating in the process evaluation provided written informed consent. All intervention participants approached to take part in the process evaluation were assured that participation was voluntary, they could withdraw from the study at any time without giving any reason, and without their medical care or legal rights being affected. Participation in the process evaluation (or not) had no bearing on intervention delivery.

### 2.5 Analysis

A qualitative approach was taken to understand the depth of individual participants’ experiences of the intervention over time, as well as practitioners’ experiences of delivery for these people.

#### 2.5.1 Within-case analyses

To understand the extent of intervention delivery for each case we first extracted the number and location of sessions from practitioner timesheet and case note data. Only sessions recorded as ‘direct participant contact’ (face-to-face, or over the telephone if the length of call exceeded ten minutes) were included. We also extracted detailed session records from the practitioner activity logs. The session descriptions were analysed and the ‘focus’ of each session was coded as one of three categories:

- Therapeutic: Record suggests the primary focus of the session was therapeutic (e.g. reflections on past behaviour);
- Practical: Record suggests the primary focus of the session was practical (e.g. housing);
- Both: Both therapeutic and practical aspects to the session were recorded.

Then we interrogated individual data sources for each case, using a combined deductive (against the coding framework based on the theory logic model) and inductive approach, being alert to unanticipated experiences.

For each case we visually itemised each component, mechanism, and outcome from the intervention theory logic model using cells in a Microsoft Excel spreadsheet. Where there was evidence of something occurring for a case we mapped it against the relevant item, colour-coding differences between where we had substantial evidence something occurred often (e.g. from multiple sources); partial, tentative or conflicting evidence (e.g. differences in accounts); or evidence that something definitely didn’t happen.

Implementation fidelity was explored by ascertaining where there were gaps or ‘silences’ in the data. Consistent silences led us to reflect on whether certain intervention components had not been delivered; and/ or whether our data capture methods had failed to identify when they had occurred. Two researchers (LW and SRB) worked independently on the analysis of each case, double-coding five cases to compare and establish coding reliability and work through uncertainties.

When all of the cases had been depicted in this way we had a visual colour-coded map of each case’s overall ‘experience’ of intervention delivery and their response to the support offer, illustrated by differences in colour-coded Excel cells. Six case study maps were presented to, and interrogated by, a sub-group of the wider project study team which included academic and practitioner representation. The six cases were selected on the basis that their participant data provided the most evidence for when mechanisms led to sustained change and when they did not.

#### 2.5.2 Cross-case analyses

Once all available evidence for each case had been analysed and coded against the relevant item from the logic model, and key issues resolved, LW and SRB conducted a cross-case comparison. Cases were grouped together, consistent with emerging patterns in outcome and mechanism activations using a ‘pile sort exercise’. We used a realist lens throughout, uncovering contingencies and conditions between component delivery, mechanism activation, outcome attainment, and (if applicable) reasons for disengagement. Both researchers conducted the cross-case comparison independently and then interpretations were brought together and considered reflexively. We discussed each case in-depth to test the rigour of assumptions made and reach consensus when differences were encountered. Concepts were considered and disconfirming ideas and alternative explanations explicitly sought. In this way, theory emerged iteratively concerning the necessary antecedents for sustained engagement and positive outcomes (see [36] for more detail on this process).

For the cross-case comparison we used Bhaskar’s (2016) ‘Description-Retroduction-Elimination-Identification-Correction’ (DREI(C)) procedural analytic method [37]. The procedure examines whether outcomes were achieved and tracks back to consider the multitude of interacting mechanisms and components of the intervention delivered to help us understand how resource offers were responded to (see Box 1 for details on the DREIC procedure).

**Box 1. Bhaskar’s (DREIC) analytic procedure**

1. *Description* of outcomes - involves noting down observations about a participant’s pathway, tracking the following: a) the resource offers made to participants through the intervention; b) were they able to achieve some of their personalised goals? c) can we see evidence of momentary, short-term effects but this does not result in the achievement of longer-term desired outcomes? d) did participants accept intervention delivery and achieve outcomes but was there little evidence of mechanism activation (e.g. feeling cared-for)?
2. *Retroduction* - assessing the evidence available and moving beyond that which was observable alone, in order to make logical inferences about underlying structures and mechanisms. Retroduction has been defined as a ‘mode of analysis in which events are studied with respect to what may have, must have, or could have caused them… Asking why events have happened in the way they did’. By interrogating the cases using retroductive reasoning we sought the answers to questions such as what led some to sustain engagement and achieve sustained change? Why did those who disengaged do so at the points that they did? What distinguished those that disengaged from those that did not?
3. *Elimination* of competing theories - entails re-examining empirical data to determine which of the competing theories best fits with the available evidence within specific cases and across cases and datasets. Tensions and ambiguities in the data were resolved through iterative discussions, though the evidence in some cases was partial and we were not able to draw a full conclusion.
4. *Identification* of specific mechanisms at work, including negative (or rival logic) effects - involves reviewing the evidence accrued and considering together: a) if sufficient evidence exists in the dataset to draw any conclusions and proceed; b) whether particular mechanisms are required to happen in sequence or parallel with other mechanisms to achieve outcomes; and c) whether there is grading of mechanism offer and emotional response.
5. *Correctio*n of initial theory and elaboration of refined theory with detailed contextual contingency in relation to operation of mechanisms - the results are compared back to the original hypotheses, the sensitizing concepts from the realist synthesis of the literature, and the descriptive statistics (where available) that detail resource offers made to individuals. We assessed where we had good evidence that particular types of support were likely to activate positive mechanism responses and lead to sustained positive outcomes.

The cross-case comparison culminated in the clustering of the 24 colour-coded case logic models into groups. We clustered cases first on the basis of patterns of intervention delivery and outcomes attained; and secondly based on hypothesised reasoning behind disengagement. Analyses were cumulative and iterative, in line with other qualitative realist evaluations [38] [39], and interpretations were explored with all stakeholders (including practitioners, other researchers, men with lived experiences of leaving prison). Post-analysis, and after the main trial quantitative evaluation, we were un-blinded to the 6-month follow-up CORE-OM scores for the 24 cases (where available). We contrasted these with their baseline CORE-OM scores and the outcomes observed from this qualitative analysis to explore similarities and differences between the quantitative and qualitative assessments of whether the intervention had produced a positive change in individual participants.

## 3 Results

### 3.1 Intervention delivery and disengagement

The within-case analyses showed that just over half the cases (13 of 24) received the minimum ‘dose’ of intervention delivery as defined for the statistical per-protocol analysis (i.e. minimum of two pre-release contacts and eight post-release contacts, see Table 1 below). Whilst all 24 cases received the minimum two pre-release contacts (M = 6.9, SD = 3.3) two cases received as little as one post-release contact (M = 12.4, SD = 11.7, range 1–47). This was despite multiple attempts to re-establish contact noted in practitioner logs. By contrast, two cases received as many as 47 post-release contacts (an average of one-two contacts per week over five-six months). Most participants received in the range of five– 15 post-release contacts, with a few dramatic outliers.

**Table 1:** Variance in intervention dose (combined pre- and post- release) delivered to depth multiple case study sample.

	Multiple case study sample (n = 24)
Number of prison-leavers in receipt of the minimum ‘dose’ of intervention delivery as intended (%)	13 (54.2)
Mean number of contacts (SD, range)	19.3 (12.1, 7–58)
Mean number of pre-release contacts (SD, range)	6.9 (3.3, 3–16)
Mean number of post-release contacts (SD, range)	12.4 (11.7, 1–47)
Number of prison-leavers in receipt of ‘met at gate’ (MAG) support (%)	21 (87.5)

Across the 24 case studies, 454 intervention delivery sessions were recorded (166 in prison and 288 in the community). A description about the purpose or content focus of these sessions was available for just 261 of these. Using the coding framework based on the theory of the logic model, the available data evidenced that the majority of sessions were ‘practical’ in nature (total n = 165, 63%) e.g. attempting to source housing, transport to/ from appointments. Only 33 sessions (13%) were coded as being solely ‘therapeutic’ in nature and a further 63 (24%) contained elements of both practical and therapeutic support. There was a general lack of evidence concerning the use of specific therapeutic techniques (such as ‘stop, rewind, explore’ or ‘microslice’) and how participants responded to these.

Once we knew the extent of intervention delivery across the 24 depth case studies, we then used the ‘DREIC’ [37] procedure and pile sort exercise described above to understand what differentiated those who completed the intervention from those who disengaged. This analyses led us to group case studies in 2 ways. First, on the basis of patterns in intervention delivery, mechanism activation, and outcome attainment; and then on the basis of patterns in reasoning underpinning disengagement from the intervention.

### 3.2 Cross-case patterns in delivery, mechanisms and outcomes

First, we sorted cases by intervention dose and content delivered, mechanisms activated, and outcomes achieved (see Table 2 below).

**Table 2:** Dose and focus of intervention sessions for case-series sample.

	Pile Sort Group					
	1	2	3	4	5	Total
	(n = 5)	(n = 4)	(n = 4)	(n = 6)	(n = 5)	(n = 24)
Number of sessions pre-release: mean	6	6	5	9	8	6.9
(SD)	(3.4)	(2.1)	(0.9)	(5.2)	(2.3)	(3.3)
min-max	3–11	3–8	4–6	3–16	4–10	3–16
Number of sessions post-release: mean	28	12	11	8	3	12.4
(SD)	(16.7)	(3.7)	(6.3)	(5.6)	(2.2)	(11.7)
min-max	10–47	8–17	5–19	2–18	1–7	1–47
Intervention session focus (group total):						
Therapeutic	7	17 *	2	5	2	33
Practical	84	19	26	22	14	165
Both	40	6	7	5	5	63
Missing session record	40	23	30	69	31	193
Total	171	65	65	101	52	454

* One participant had 14 therapeutic sessions.

We found that just five of the 24 cases sustained positive change on the logic model outcomes (pile sort group 5). These cases received the greatest number of intervention sessions post-release (M = 27, SD = 16.9, range 9–46) and the greatest number of therapeutic focused sessions (M = 7.8, SD = 5.1, range = 3–16). By contrast, the other 19 cases disengaged before the end of the intervention and did not sustain positive change. On average they received 7.5 sessions (SD = 5.5, range 1–18) and had fewer therapeutic focused sessions (M = 3.3, SD = 4.6, range 1–16).

A detailed summary of the variations between pile sort groups in terms of intervention delivered, mechanisms activated and outcomes achieved can be found in Table 4 in S3 File. In brief, the evidence suggested that the cases in pile sort group 5 maintained contact with their Engager practitioner and achieved sustained change based on their jointly agreed individualised goals, which included not returning to prison, abstaining from substances, gaining employment, and/ or improving familial relationships. The extent of therapeutic support appeared to differentiate those in this group (who sustained change) from those in groups 1–4 (who did not sustain change). None of the cases who had not engaged appeared to be doing well. These two contrasting experiences are illustrated by the statements below. All participant names have been changed:

*“I found it really useful, taking me to probation and helping me to keep to appointments and, you know, being able to be open and honest and talk to him about other things that have gone on and happened and things. So things are really good actually… Staying clean, staying on the right track”*

Michael, six-month follow-up

*“Didn’t see [the practitioner] many times, like three, four, maybe. Everything just sort of went downhill when I moved in [to the shared house] really because that’s when I started selling drugs”*

Anthony, six-month follow-up

### 3.3 Cross-case patterns in reasoning behind sustained and non-sustained engagement

The content of intervention delivery appeared to differentiate those who sustained change from those who did not, so we then used the DREIC [37] procedure again to look at why cases engaged or disengaged from the intervention, exploring elements of delivery by practitioners, participant response and context. Through the data we interpreted both the level of engagement and the perceived ‘state’ participants were in over the course of the intervention. After doing this, we grouped the 24 cases according to the particular state they were in when they finished the intervention (either through completion or disengagement). Analysis of the groups of cases led to the conceptualisation of five ‘internal states’, representing a data-grounded theoretical explanation of why individuals maintained engagement or disengaged base on how their individual needs were, or were not, adequately met by the intervention. The conceptual states were named: ‘Crises but coping’, ‘Crises and chaos’, ‘Resigned acceptance’, ‘Honeymoon’ and ‘Wilful withdrawal’. They aim to represent the states of thinking, emotion and behaviour each individual was in, in terms of continued engagement or disengagement from the intervention, at the end of intervention delivery. The five states and their distinguishing features are summarised below in Table 3. Worked exemplars illustrating how we arrived at our conclusions can be found in S4 File.

**Table 3:** Summary of the five internal states and distinguishing features.

Internal state	Distinguishing intervention delivery features	Description of internal state and effect on intervention disengagement	Exemplar quote from representative case
Crises but coping	Received the intended intervention in terms of dose and content. Substantial therapeutic content throughout post-release phase, cases maintained engagement despite ongoing personal challenges.	Did not disengage. By the end of the intervention, these cases were in a state characterised as ‘crises but coping’. They maintained engagement with the Engager practitioner through personal and social hardships (e.g. substance misuse issues and/ or homelessness). By developing and acting on a shared understanding of needs and goals, they developed a capacity to mentalise, increase self-agency to change behaviour, and sustain motivation towards goals. They learnt to make positive choices in stressful situations rather than responding to crises in ineffectual ways. This distinguishes these cases from the trajectories of the other cases who did not sustain engagement in moments of crises and were unable to reach this state of ‘crises but coping’.	*“I learned how to talk about things*. *Before I wouldn’t have even spoken about this [relapse]*, *I would have just sat here quiet*. *[Before the practitioner] I’d never really had anyone say how are you doing*? *How has that impacted you*? *How has that made you feel*? *So I suppose now I’m able to talk to people a lot better instead of it all getting pushed to one side”*
			Michael (‘Crises but coping’ case at 12 month follow up)
Crises and chaos	There was some early development of a shared understanding regarding hopes and goals but may not have received sufficient therapeutic support to increase their capacity to learn how to mentalise, regulate emotions and develop self-agency	These prison-leavers experienced significant challenges on release and were unable to overcome them. Unlike those experiencing ‘crises but coping’, they became overwhelmed by their circumstances and believed their situation to be inescapable. On release from prison they experienced challenges that undermined their wellbeing and they descended into chaotic thinking patterns. Practitioners were not able to encourage mentalisation and these prison-leavers were generally unable to maintain any type of contact with services, including Engager, achieving very few medium-term positive outcomes.	*“I thought that there was not a lot of difference that you lot could make*. *Every day I was getting suicidal thoughts*. *Every pay day I was thinking about buying lots of heroin and ODing*. *Cos I didn’t see no end to it*. *It’s all going to hell*. *If I hadn’t have Od’d I would have purposely done it myself anyway”*
			James (‘Crises and chaos’ case at 12 month follow up)
Resigned acceptance	These prison-leavers were appreciative of support, initially maintaining some engagement with the Engager practitioner with whom they had good rapport, achieving steps towards goals in the short-term. However, when faced with familiar challenges on release their belief in themselves to make changes waned.	Cases with resigned acceptance disengaged while reasoning their circumstances were inevitable and unchangeable. An absence of discernible distress distinguished these cases from those in ‘crises and chaos’, which appeared to be a protective mechanism to prevent them experiencing more heightened emotions. ‘Resigned’ to life as it always had been, they disengaged, slipping back into old behaviour patterns. This was not addressed through appropriate therapeutic support by the Engager practitioner, due to a gap in trust in the practitioner’s ability to mobilise resources to support them achieving their goals, and increase their self-esteem.	*“I go back and forth from mates’ houses [to sleep]*. *To be honest*, *that’s not too good to but you know*. *I’m coping*, *I’m managing sort of*. *It’s stressful sometimes but other than that*, *it’s alright*. *I’ve always got somewhere to get me head down*, *I’m getting by”*
			Adam (‘Resigned acceptance’ case at 6 month follow up)
Honeymoon	These cases were open and content to engage with the Engager practitioner while in prison, often having good rapport. Post-release they quickly discontinued contact with the practitioner, contending they were no longer in need.	Honeymoon prison-leavers confidently projected the image that they were mastering the trajectories of the lives. They appeared self-reflective and articulate about where things had gone wrong in the past; naively confident about their up-coming release despite lacking a well-developed plan. They had some forms of stability (e.g. housing/ job opportunities) which masked unaddressed vulnerabilities. Enduring challenges or a series of obstacles soon overwhelmed their façade of coping, and they regressed into old patterns of behaviour, unable to mentalise effectively. For honeymoon cases Engager practitioners were unable to find an appropriate ‘angle’ through which to address the unrealistic optimism these prison-leavers had in the potential for their existing resources to fulfil all their needs. Practitioners tended to take cases’ assertions they were managing well at face value, not spending sufficient time developing trust so that they felt safe enough to be vulnerable and open up about their concerns.	*“When I come out last time I got back on the drink and my life just went downhill again*. *It’s made me realise again that alcohol’s one of my main triggers to committing crime*. *So I’ve gotta stay off the drink because I can’t control it*, *the drink controls me and I end up doing crazy things*. *So at least I’ve made a big step now though and I’ve realised that”*.
			Sam (‘Honeymoon’ case at his 6 month follow up interview*)
			*Soon after release Sam found employment and disengaged from Engager because he was feeling confident about his independence. *However within 8 months of his release he had returned to using alcohol*, *lost his job*, *and had attempted suicide*.
Wilful Withdrawal	Wilful withdrawal prison-leavers actively resisted the intervention whilst still in prison, or withdrew from it immediately on release. They tended to decline support early on, were reluctant to take up opportunities the practitioner arranged for them, and unwilling to do any therapeutic work.	Practitioners were not able to establish trust with these prison-leavers. This meant that shared understandings were not developed together and Engager practitioners were unable to sustain contact after release as there was no rapport between them.	*“I saw [the practitioner] once in town but that was unplanned*, *I just happened to see them*. *I didn’t want to get in touch with them*, *I didn’t want the help*, *I stopped listening*. *I wanted to carry on working and [the practitioner] offered to help me get work but I didn’t want the support*. *I don’t really know what Engager is”*
			Liam (‘Wilful withdrawal’ case at his 6 month follow up interview)

There were no discernible contextual differences between the five ‘crises but coping’ cases who sustained engagement, and the other 19 who did not. The former were no less in need of support in terms of substance use, homelessness and broken relationships and did not necessarily display motivation or confidence in their capacity to change at the outset, but each went on to build trust, maintain engagement with the EP, and achieve positive change. The key determinants of sustained engagement and positive outcomes were identified as:

- The quality of the relationship between practitioner and participant–needed to be based on genuine concern, the building of epistemic trust, unconditional positive regard, and demonstrations of integrity over time;
- The quality of therapeutic work undertaken–needed to address personal goals, and support the participant’s development of confidence and self-belief that their goals were attainable; and
- The presence of a robust shared understanding between practitioner and participant–needed to be genuinely understood by both parties and based on the participant’s own priorities, rather than a generalised assumptions of need.

Importantly, achievement of medium- to long-term outcomes was associated with mechanisms which had been activated more than once or twice, suggesting prison-leavers needed a consistent, tailored intervention, grounded in quality therapeutic content and delivered over time in order to have a sustained effect.

*“When you’ve got someone telling you you’re worthless all the time and it’s your fault all the time you start to believe it so you think you are worthless*. *Until someone says*, no Callum*, *you’re actually alright… Getting the help from you guys and that*, *you’re telling me not to give up sort of thing*, *it does help*. *Like*, *I’ve changed the way I’m thinking a little bit*. I don’t need to press that f*** it button too quickly because otherwise I’ll just end up back in prison. *Housing is probably the main priority at the minute*, *and the Jobcentre*, *show someone I’ve got income coming in… I’m more positive about myself [now]”* Callum, a ‘crises but coping’ participant, at his 12-month follow-up interview

Lastly, we explored how the qualitative analysis outcomes for these cases compared with the change in their CORE-OM scores from baseline to six-month follow-up (where data was available). We found that our interpretation of what participants and practitioners experienced as a ‘success’ was not always reflected in the change to CORE-OM scores (see additional file 3). Understanding participants’ individual contextual circumstances and ‘internal states’ during the intervention can go some way to explain this discrepancy. As an example illustration, a participant we characterised as being in a ‘honeymoon’ state during early disengagement reported responses to questions in relation to his mental health which meant he had a significantly improved 6-month follow-up CORE-OM score. However, by the time he was interviewed again two months later, he had lost his job, his mental health had regressed, he was back self-medicating, self-harming, and had made a further suicide attempt.

By contrast, a participant we identified as being in a ‘crises but coping’ state at the end of the intervention reported that he was still experiencing a lot of anxiety post-release. This participant reported a non-clinically significant improvement in his six-month follow-up CORE-OM score, but he himself identified that his anxiety was directly related to his ongoing complex circumstances. He was in the process of removing himself from a toxic relationship, continuing to abstain from substances, had not re-offended, and as a result of the therapeutic work with the Engager Practitioner, was managing uncomfortable thoughts and feelings for the first time instead of blocking them out as he had done in the past. While this participant’s overall change in CORE-OM score was negligible, and housing status worse, his personal circumstances from baseline to follow-up were vastly different and he saw himself as having made several sustained positive changes to the trajectory of his life.

### 3.4 Implementation fidelity

A thorough examination of delivery fidelity to the theoretical model for the whole trial sample will be reported elsewhere. During the analyses reported here, we recorded gaps in intervention delivery for individual cases and then compared patterns of delivery fidelity across cases. We identified evidence of common gaps in certain intervention components being delivered across-cases. These are summarised below.

#### 3.4.1 Overarching site differences

Team dynamics encountered difficulty where Engager practitioners had different visions of how the intervention should be delivered, for instance, one Engager practitioners’ sense of ‘going the extra mile’ for a prison-leaver, could be interpreted by another as fostering dependency. Risk formulations were fallible as supervisors had different perceptions of risk between the two sites. In one site there was a tendency towards overreliance on historical presentation of risk rather than dynamic present factors. When this translated to there being a requirement for prison-leavers to be seen by two practitioners together, this negatively affected the amount of contact they received. We saw variation between sites in terms of team supervision records and the level of information sharing and planning that took place when an Engager practitioner took a leave of absence. Although they covered for each other at times, in some cases there seemed little information sharing about the particular cases involved meaning that some prison-leavers missed out on valuable sessions because stand-in practitioners weren’t able to progress with any ongoing work. This lack of continuity (in terms of both practitioner and session content) may also have affected prison-leavers’ ongoing engagement because they tended to placed great value on continuity of care. The geographical dispersal of prison-leavers in one site, who were often located in rurally isolated villages, meant that liaison work with other services was more challenging. Similarly, the wider geographical area one team were working in made it more difficult to find a convenient location to meet regularly as a team.

#### 3.4.2 Therapeutic emotional work

We lacked evidence concerning the use of MBA techniques with prison-leavers. Engager practitioners often weren’t readily able to recall specific examples of times they’d used MBA, and their session note records tended to centre on what the prison-leaver said rather than the therapeutic tools they themselves used to elicit responses. This absence of therapeutic work, and a related lack of shared understanding between practitioner and prison-leaver, appears to underpin disengagement from the intervention for a number of cases. In some instances over the course of the intervention the practitioner appeared to have learnt to rely on generalised assumptions of need pertaining to ‘prison-leavers in general’, such as housing or substance misuse support, rather than ascertaining from the prison-leaver himself what support he might value. Where mutually-agreed upon goals weren’t specified before a prison-leaver’s release, and achievable steps to attain said goals under-developed, the prison-leaver discontinued engagement with the intervention and their capacity to sustain positive change was undermined.

#### 3.4.3. Maintaining community contact

Sustaining contact with prison-leavers post-release was another area of difficulty for practitioners. When a prison-leaver was released to some form of housing (including private, temporary or supported), practitioners had a direct line of contact to them after their release; and this remained true even when they didn’t have a telephone or weren’t attending probation. However often prison-leavers were released to no fixed abode, with no personal telephone, and no requirement to attend probation regularly. When this happened the practitioner struggled to find a means of contacting the prison-leaver and by the time they did, the prison-leaver was often back in prison after returning to maladaptive patterns of behaviour, and their time on the intervention had elapsed.

#### 3.4.4. Family and friends liaison

Only in a handful of cases did practitioners have direct contact with family and friends. Only in one or two cases did the prison-leaver’s family feel directly supported by the practitioner. Our implementation data suggests that practitioners saw contact with the family as something which helped offer another perspective on the prison-leaver, and a context for understanding of some of their issues and challenges. Contact with the family meant the practitioner could better assess the support the prison-leaver could draw on, or the dysfunctional and tenuous relationships which might undermine their success in the community. Work around family and friends may be less direct and more about emotional work with the prison-leaver to help them navigate their way through difficult relationships. This component perhaps wasn’t always apparent in our data because the prison-leaver had little or no familial or other support to draw from. Prison-leavers sometimes would talk about regaining contact with family and on other occasions they would explain that there had been irreparable damage and familial ties had been severed.

#### 3.4.5. Handling a good ending to the intervention

So many of the cases disengaged before they reached the end of the intervention so establishing whether there was consistency in how prison-leavers experienced the ending was difficult. Clear documentation regarding the transfer of support onto another worker was evident in only 3 of the 24 cases we looked at; and in all 3 cases the contact arrangements were often loose and short term (for example support while living in temporary accommodation). In some cases, either there was no ongoing services working with the prison-leaver, or there was no identifiable key worker that the practitioner could assign their case too. For others, they had disengaged earlier on and didn’t have a formal ending. This absence of appropriate hand-over support lends itself to the idea of looking for cost-effective ways to ‘leave the door open’ past the end of the intervention so that prison-leavers aren’t left in a void of support.

The standards for reporting qualitative research (SRQR) was used to guide the reporting of qualitative findings (see S5 File).

## 4 Discussion

The findings revealed that around half the sampled cases received the minimum ‘dose’ of intervention delivery; and although practical support was delivered on many occasions, this was insufficient to generate sustained positive change for many participants. The most profound changes were observed in participants who sustained engagement and received more therapeutic support; reaching a state of ‘*crises but coping’*. They had developed their capacity to think, and to understand their circumstances while striving to improve them. By comparison, the other participants, who had less therapeutic support and disengaged before they had developed their capacity to mentalise effectively, did not achieve the same status of ‘coping’.

‘Crises and chaos’ prison-leavers, despite initial positive responses, were unable to self-regulate their emotions and found it difficult to think clearly about how to effect change. Those with ‘resigned acceptance’ may be understood as feeling ‘hopeless’, defined as a system of belief characterised by negative expectations for the future; and perceived helplessness in one’s own capacity to change such anticipated outcomes [40, 41]. Similarly, ‘wilful withdrawal’ prison-leavers felt unable to confront their difficulties, opting to actively ‘escape’ and return to previous coping behaviour patterns. By contrast, ‘honeymooners’ displayed an apparent initial competence to cope with life which masked the true insecure reality of their situations. This was further compounded by an inability to communicate their vulnerability to others, resulting in their needs going unheard and their circumstances unchanged.

### 4.1 Understanding intervention disengagement

To engage in and benefit from treatment, offenders may need to possess certain cognitive, emotional, volitional, and behavioural properties, and exist in contexts where changes are possible and supported [21, 42–44]. For participants unable to sustain engagement, there were incompatibilities between their internal state, the intervention, and the external context at the point they took part. ‘Crisis’ was not a predictor of disengagement. Indeed, the five ‘crises but coping’ prison-leavers were still experiencing substantial social crises after the conclusion of the intervention. This demonstrates that, when delivered well, it was possible to achieve change in this population within this setting. Ensuring delivery fidelity was, however, an ongoing challenge. As reported above, therapeutic emotional work was often observed to be missing or inconsistently delivered. The psychological aspects of delivery which appeared to be most prone to delivery failure, in what were extremely challenging conditions for practitioners, were developing a true ‘shared understanding’ and routinely applying mentalisation based approaches.

To the best of our knowledge, the ‘crises but coping’ participants had no discernibly distinguishable features at recruitment to the trial. Their social environments, offending history, personal circumstances, and motivation to change, were similar to the other participants. It is plausible that participants who were categorised as displaying chaos, honeymoon, resigned acceptance or wilful withdrawal characteristics might have been able to achieve a ‘crises but coping’ state if they had received more, and better, therapeutic support. We were not able to measure all potential contextual features affecting disengagement. However, while reasons behind participants’ disengagement were highly personal and individual, each disengagement reflects a failing in the intervention theory and/ or delivery to adequately address their needs.

Through understanding the circumstances that led to disengagement, we firstly proposed enhancements to make the intervention more implementable. For example, the therapeutic mentalisation-based approach was intended to underpin all elements of delivery including building trust and rapport, developing a shared understanding, co-creating a shared action plan and mobilising resources to support meeting goals. However, our analysis demonstrated that delivery of ‘therapeutic’ support was often lacking or artificially delineated from the rest of the intervention in ways that were not intended. This highlights the need for clear and accurate operationalisation of an intervention theory, transmitted effectively to those responsible for delivery, in order to ensure that misinterpretations do not lead to inconsistent or flawed delivery. Further enhancements to training and supervision in terms of quality, intensity and frequency may also be required when supporting experienced but clinically unqualified practitioners in complex environments.

Secondly, aspects of the intervention theory may need to be improved. Practitioners need to be equipped with varied skills and methods of approach to re-engage participants from diverse groups whose commitment to an intervention may vary over time. Good therapeutic work is essential for sustained engagement and extended contact time within the protected structure of the prison environment may provide a valuable opportunity for practitioners to build trust and develop a true shared understanding with the participant. For example, multiple prison contacts offer practitioners the opportunity to get beneath the ‘honeymooner’ façade of coping, and time to demonstrate trustworthiness to those at risk of ‘wilfully withdrawing’. Prison contacts may also provide practitioners with an opportunity to develop a sense of control in someone with ‘resigned acceptance’ and can help build the thinking capacity of a ‘crises and chaos’ prison-leaver before they are faced with pressures of living in the community and associated heightened unregulated emotions. Dealing with such a range of psychological challenges was not fully anticipated and we propose that the theory, manual and training should reflect the knowledge and skills needed to support the range of internal states likely to be encountered.

### 4.2 Challenges of evaluating complex interventions in complex systems

Delivering complex person-centred interventions with fidelity (as intended, with engagement) [45–48] has repeatedly been shown to be hard to achieve [49–52]. Deviations from adherence to the intervention model are particularly likely in cases like Engager, whose model is broad in scope, non-linear in nature, and includes multiple component parts [7]. Interventions situated within wider complex social systems raise further challenges, because of the multitude of interdependent elements which can affect each other and the system as a whole [53]. The criminal justice system itself may be considered one of the most complex systems to conduct research in [54].

Mapping out intervention components is a method of increasing robustness of fidelity testing, a process adopted in this study. However, the intervention’s flexible, person-centred, structure meant that component delivery was not intended to be uniform across the study population and the absence of certain components may have resulted from deliberate omission rather than non-adherence to the model. Our case study approach, with data covering multiple perspectives over time, facilitated interpretation as to whether intervention delivery occurred and was responded to as intended. Limitations included the relatively small number of cases and inevitable attrition of prison-leavers from the research process resulting in some partial case studies and incomplete sources of evidence. This highlights a particular evaluation difficulty in terms of delineating between evidence of limited delivery versus limited evidence of delivery.

This process evaluation needs to be considered alongside the other analyses forming the parallel process evaluation and quantitative evaluation of the RCT. Additionally our depth case study approach raises important questions about the value and reliability of standard outcome measures such as the CORE-OM in the evaluations of complex interventions. It has been argued that such measures may be considered inadequate in complex intervention evaluations because they do not account for individual contexts, subjective perceptions of success, or unpredictable idiosyncratic individualised changes arising from person-centred interventions [55, 56]. For example, in this sample of Engager participants some demonstrated no ‘clinical recovery’ in their psychological distress, according to the Engager primary trial outcome measure [33] and yet appeared to be to some extent on the path to recovery in the process evaluation; for others the reverse was true. ‘Recovery’ defined as ‘a deeply personal, unique process of changing one’s attitudes, values, feelings, goals, skills, and/ or roles’ [57] is highly subjective and variable, so perhaps the discrepancy is not surprising. As such, we suggest that outcome measures such as the CORE-OM may provide a snapshot image of how participants are in a particular moment in time, but they do not always reveal the underlying complexity of an individual’s response to an intervention. The process of change and development of resilience may not necessarily be captured in discrete trial outcome measures, and here the CORE-OM may not have been sensitive enough to detect the early changes in participants’ thought processes and coping strategies during the relatively short time frame of the study. Understanding the multi-faceted nature of individuals’ responses to an intervention is crucial for understanding how to optimise its future implementability; and we would argue also provides an alternative lens on effectiveness.

## 5 Conclusions

This paper demonstrates that understanding delivery of a complex intervention, and participants’ responses to its component parts, provides useful lessons in how to make person centered (flexible) complex interventions implementable. The qualitative methodology provided deeper insights and understanding than the more binary measures of efficacy recorded in the quantitative evaluation of the RCT. This demonstrates the limitations of relying on, or prioritizing, quantitative outcome measures when assessing the success of intervention implementation. By examining programme theory in relation to delivery we found that rapport, while a necessary precursor for therapeutic work, was not a guarantee of longevity of Engager practitioner and participant relationships post-release. Core delivery mechanisms often had to be ‘triggered’ numerous times to produce sustained change. In addition, implementation failure in the shape of inconsistent or missing therapeutic work, brought with it missed opportunities for change. By contrast, when delivered as intended with a robust shared understanding developed using a mentalisation-based approach, some cases demonstrated positive sustained change while managing ongoing personal and social difficulties. Prior motivation to change was not found to be a pre-requisite for success; individuals’ readiness and commitment to change ebbed and flowed. Thus compatibility between practitioner, participant, and setting are continually at risk of being undermined by implementation failure as well as changing external circumstances and participants’ own weaknesses. Furthermore, implementation of person centred interventions are at risk of failing, if practitioners do not adequately continually adjust to individuals’ needs. To address this, and change from a requirement for standardised delivery, practitioner teams are likely to require enhanced training and regular robust clinical supervision to help them sustain good practice. Implementation of complex care interventions to those with even modest levels of distrust must account for and connect to participants’ priorities, grounded in a relationship underpinned by epistemic trust, in order for them to be more widely implementable.

## Supporting information

S1 File Engager programme theory tube map. (DOCX)

S2 File Process evaluation participant data sources. (DOCX)

S3 File Patterns in delivery. (DOCX)

S4 File Patterns in disengagement. (DOCX)

S5 File SRQR checklist. (DOCX)

## Abbreviations

CORE-OM: Clinical Outcomes in Routine Evaluation- Outcome Measure
FPE: Formative Process Evaluation
M: Mean
MAG: Met-At-Gate
MBA: Mentalisation-Based Approach
PE: Process Evaluation
RCT: Randomised-Controlled Trial
SD: Standard Deviation
SRQR: Standards for Reporting Qualitative Research
