# Supplementary figures and images for: Interrogating intervention delivery and participants’ emotional states to improve engagement and implementation: A realist informed multiple case study evaluation of Engager
Source: PLoS One. 2022 Jul 14;17(7):e0270691. doi: 10.1371/journal.pone.0270691 (PMC9282559; doi:10.1371/journal.pone.0270691)

S1 File: The Engager Intervention Programme Theory Tube Map


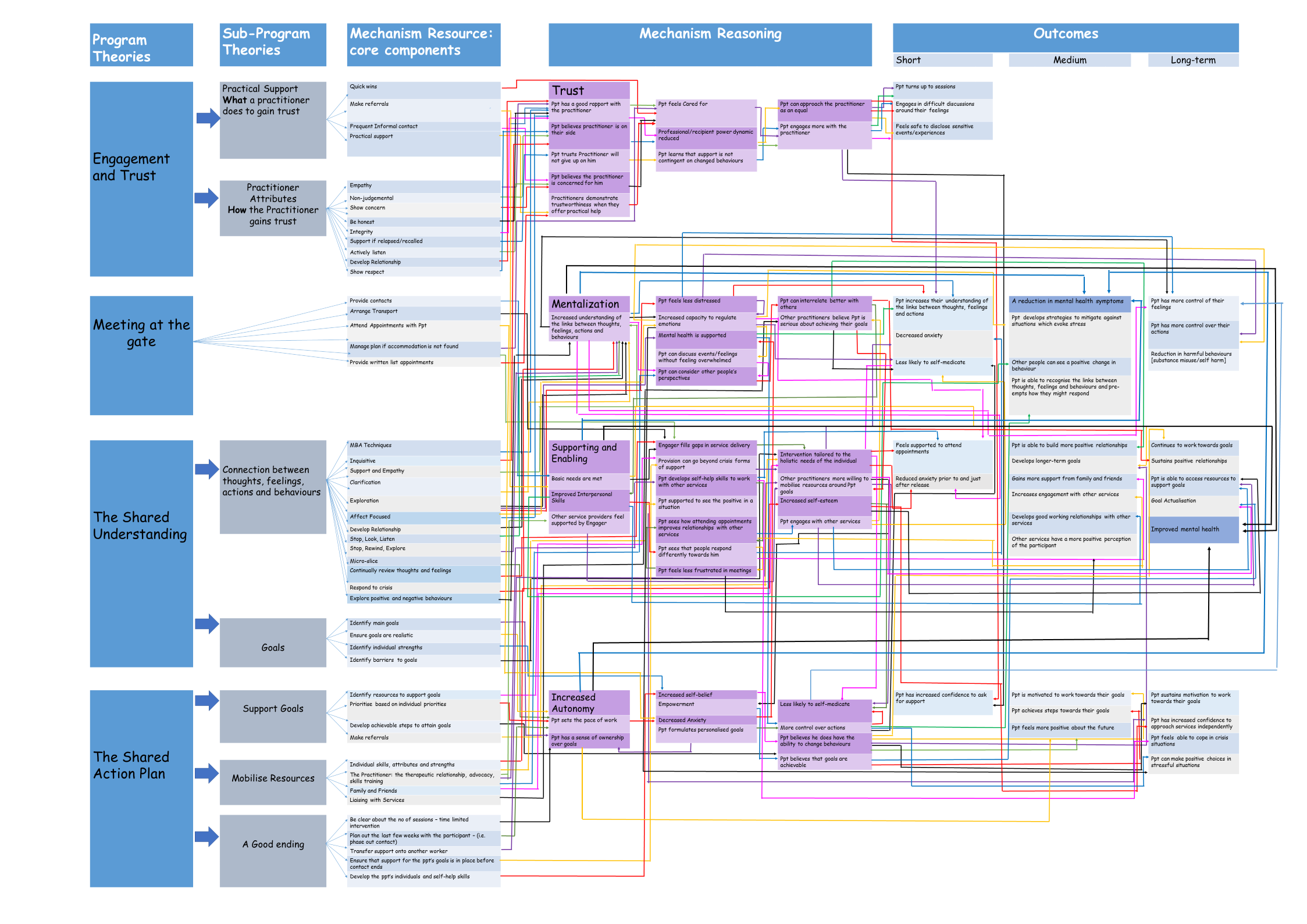

Supplement: S1 File — (DOCX) [file pone.0270691.s001.docx]
