# Supplementary material for: Interrogating intervention delivery and participants’ emotional states to improve engagement and implementation: A realist informed multiple case study evaluation of Engager
Source: PLoS One. 2022 Jul 14;17(7):e0270691. doi: 10.1371/journal.pone.0270691 (PMC9282559; doi:10.1371/journal.pone.0270691)
Supplement: S2 File — (DOCX) [file pone.0270691.s002.docx]

S2 File. Overview of the sources of data collected for each participant in the depth multiple case study analysis

Table 1. Sources of data collected for each participant case

|  | **Participant Number** | | | | | | | | | | | | | | | | | | | | | | | |
| --- | --- | --- | --- | --- | --- | --- | --- | --- | --- | --- | --- | --- | --- | --- | --- | --- | --- | --- | --- | --- | --- | --- | --- | --- |
| **Data Source** | **1** | **2** | **3** | **4** | **5** | **6** | **7** | **8** | **9** | **10** | **11** | **12** | **13** | **14** | **15** | **16** | **17** | **18** | **19** | **20** | **21** | **22** | **23** | **24** |
| Practitioner timesheet record of activities | x | x | x | x | x | x | x | x | x | x | x | x | x | x | x | x | x | x | x | x | x | x | x | x |
| Session records | x | x | x | x | x | x | x | x | x | x | x | x | x | x | x | x | x | x | x | x | x | x | x | x |
| Semi-structured interview with participant at baseline | x | x | x | x | x | x | x | x | x | x | x | x | x | x | x | x | x | x | x | x | x | x | x | x |
| Semi-structured interview with participant at follow-up (3-6 months post-release) | x | x | x | x | x | x | x | x | x | x | x | x | x | x | x | x | x | x | x | x | x | x | x | x |
| Semi-structured interview with participant 12 months post-release | x |  |  |  |  |  |  |  |  |  | x |  |  |  |  |  |  | x |  |  |  |  |  |  |
| Semi-structured interview with Engager practitioner | x | x | x | x | x | x | x | x | x | x | x | x | x | x | x | x | x | x | x | x | x | x | x | x |
| Semi-structured interview with practitioners from other services | x | x | x |  |  | x |  |  |  |  | x |  | x |  | x |  |  | x |  |  | x | x |  |  |

Whilst it is challenging to gain complete data sets, deeper understandings can be gained by interrogating as many data sets as available within and across the cases.
