# Supplementary material for: Interrogating intervention delivery and participants’ emotional states to improve engagement and implementation: A realist informed multiple case study evaluation of Engager
Source: PLoS One. 2022 Jul 14;17(7):e0270691. doi: 10.1371/journal.pone.0270691 (PMC9282559; doi:10.1371/journal.pone.0270691)
Supplement: S3 File — (DOCX) [file pone.0270691.s003.docx]

S3 File: Patterns in delivery, mechanisms and outcomes

Table 4. Case-series sample grouped according to dose and content of intervention delivered and outcomes achieved and sustained

|  | **Salient variations in intervention delivery** | **Intervention components delivered** | **Mechanisms activated** | **Intervention outcomes achieved** |
| --- | --- | --- | --- | --- |
| **Group 1** | *Trust & Engagement:*  Trust often initiated by practical quick win (e.g. service referral) but engagement sustained by an ongoing non-judgemental and empathetic stance, consistency in approach, persistence (finding new ways to engage), and a shared understanding. Substantial trust necessary for sustained engagement  *Meeting at the Gate:*  Three of the five participants were met at the gate, transported to their release area, accompanied to key appointments, and (where applicable) were supported with a plan when accommodation was not found for the first night. One participant was met at probation and supported through this appointment; and the other declined the offer of release day support.  *A Shared Understanding:*  Solid trust was a pre-requisite for emotional shared understanding to develop. A shared understanding was then essential for securing ongoing engagement and confidence to affect change. Participants were supported through relapse/ recall and these crises supported the ongoing refinement of their mutual shared understanding.  *The Shared Action Plan:*  Main goals, strengths, resources, and risks were identified and achievable steps prioritised. Provision of contacts and ongoing liaison and advocacy work between key service personnel, and (where possible) participants’ family/ friends to strengthen support system and support attainment of goals. | *Trust & Engagement:*  Quick wins, referrals, frequent informal contact, practical support, shows empathy, non-judgemental, shows concern, honest, demonstrates integrity, supports if relapsed/ recalled, actively listens, develops relationship, shows respect.  *Meeting at the Gate:*  Arranges transport, attends appointments, manages plan if accommodation not found.  *A* *Shared Understanding:*  Uses mentalisation techniques, inquisitive, shows support and empathy, uses clarification and exploration, affect-focused, continually reviews thoughts and feelings, responds to crises, explores positive and negative behaviours, identifies main goals, ensures goals are realistic, identifies individual strengths, identifies barriers to goals.  *The Shared Action Plan:*  Identifies resources to support goals, prioritises goals on individual priorities, develops achievable steps to attain goals, makes referrals, liaises with family and friends, liaises with services, plans out the last few weeks of support i.e. phase out contact, ensure support for the participant’s goals in place before ending, develop the participant’s individual and self-help skills. | *Trust & Engagement:*  Has a good rapport with the practitioner.  Believes the practitioner is on their side/ concerned for him.  Feels the practitioner demonstrates trustworthiness when they offer practical help.  Feels cared for.  Professional/ recipient power dynamic reduced.  Learns that support is not contingent on changed behaviours.  Can approach the practitioner as an equal.  Engages more with the practitioner.  *Mentalisation:*  Increased understanding of the links between thoughts, feelings, and behaviour.  Feels less distressed.  Increased capacity to regulate emotions.  Mental health is supported.  Can discuss events/ feelings without feeling overwhelmed.  Can interrelate better with others.  *Supporting & Enabling:*  Improved interpersonal skills.  Provision can go beyond crisis forms of support.  Develops self-help skills to work with other services.  Supported to see the positive in a situation.  Sees how attending appointments improves relationships with other services.  Feels less frustrated in meetings.  Increased self-esteem  Engages with other services.  *Increased autonomy:*  Sets the pace of work.  Has a sense of ownership over goals.  Increased self-belief.  Empowerment.  Decreased anxiety.  Formulates personalised goals.  More control over actions.  Believes he does have the ability to change behaviours. | *Short term:*  Turns up to sessions.  Engages in difficult decisions around their feelings.  Feels safe to disclose sensitive events/ experiences.  Increased understanding of the links between thoughts, feelings and actions.  Decreased anxiety.  Less likely to self-medicate.  Feels supported to attend appointments.  Reduced anxiety prior to and just after release.  Increased confidence to ask for support.  *Medium term:*  A reduction in mental health symptoms.  Recognises links between thoughts, feelings and behaviours and pre-empts how they might respond.  Develops strategies to mitigate against situations which evoke stress.  Develops good working relationships with other services.  Increases engagement with other services.  Achieves steps and sustains motivation towards goals.  Able to access resources to support goals.  Feels more positive about the future.  *Longer term:*  Has more control over their feelings and actions.  Reduction in harmful behaviours (substance misuse/ self harm).  Can make positive choices in stressful situations.  Sustains motivation and continues to work towards goals.  Sustains positive relationships.  Able to access resources to support goals.  Goal actualisation.  Increased confidence to approach services independently.  Feels able to cope in crisis situations.  **No. of pts with improved CORE-OM score at 6 month follow up: 2** |
| **Group 2** | *Trust & Engagement:*  Trust built through practical quick wins, and non-judgemental and affect-focused approach to interactions. Initial engagement post-release (e.g. attending appointments together) and support during momentary crises (e.g. brief relapse). Outside events, existing crises (e.g. longstanding substance addiction), and limited therapeutic components from Engager Practitioner meant that engagement waned after the first few weeks/ months post-release.  *Meeting at the Gate:*  All four participants were provided with release day support, two were met at the prison gate and two at probation.  *A Shared Understanding:*  Some evidence of a shared understanding with participants making connections between how their thoughts, feelings, and actions were interlinked. The depth of trust between practitioner and participant may have been fairly shallow, as it did not prevent disengagement at crucial points of need.  *The Shared Action Plan:*  Main goals were identified and support needs prioritised. Engager practitioners liaised with services and attended appointments with the participants throughout the course of the their engagement. | *Trust & Engagement:*  Quick wins, referrals, frequent informal contact, practical support, shows empathy, non-judgemental, shows concern, honest, demonstrates integrity, actively listens, develops relationship, shows respect.  *Meeting at the Gate:*  Arranges transport, attends appointments, manages plan if accommodation not found.  *A* *Shared Understanding:*  Uses mentalisation techniques, inquisitive, shows support and empathy, uses clarification and exploration, affect-focused, continually reviews thoughts and feelings, identifies main goals.  *The Shared Action Plan:*  Identifies resources to support goals, prioritises goals on individual priorities, develops achievable steps to attain goals. | *Trust & Engagement:*  Has a good rapport with the practitioner.  Believes the practitioner is on their side/ concerned for him.  Feels the practitioner demonstrates trustworthiness when they offer practical help.  Feels cared for.  Professional/ recipient power dynamic reduced.  Learns that support is not contingent on changed behaviours.  Can approach the practitioner as an equal.  Engages more with the practitioner.  *Mentalisation:*  Increased understanding of the links between thoughts, feelings, and behaviour.  *Supporting & Enabling:*  Provision can go beyond crisis forms of support.  Supported to see the positive in a situation.  Feels less frustrated in meetings.  Increased self-esteem  *Increased autonomy:*  Sets the pace of work.  Believes he does have the ability to change behaviours [short-term only; unsustained]. | *Short term:*  Turns up to sessions.  Engages in difficult decisions around their feelings.  Feels safe to disclose sensitive events/ experiences.  Increased understanding of the links between thoughts, feelings and actions.  Feels supported to attend appointments.  Reduced anxiety prior to and just after release.  Increased confidence to ask for support.  *Medium term:*  Recognises links between thoughts, feelings and behaviours and pre-empts how they might respond.  Develops strategies to mitigate against situations which evoke stress.  Less likely to self medicate [only in the short term; unsustained].  *Long term:*  n/a  **No. of pts with improved CORE-OM score at 6 month follow up: 2** |
| **Group 3** | *Trust & Engagement:*  Trust was built primarily through offers of practical support pre-release, and was ‘superficial’ in terms of emotional depth. Participants sustained engagement for just a short amount of time post-release (e.g. meeting Engager practitioner for initial community session and then dropping out of contact).  *Meeting at the Gate:*  All four participants were provided with release day support, two were met at the prison gate and two at probation.  *A Shared Understanding:*  Limited emotional depth to the rapport between Engager Practitioner and participant and little evidence of work into the recognition of links between thoughts, feelings and actions. Participants guarded or had a naïve optimism in their own capacity and social capital to cope.  *The Shared Action Plan:*  Main goals were identified but tended to be based on generalised assumptions of need rather than things the participants themselves wanted support with. Some liaison with services and Engager practitioner attended appointments with the participants for the limited time they engaged. | *Trust & Engagement:*  Quick wins, practical support, shows empathy, non-judgemental, shows concern, honest, actively listens, shows respect.  *Meeting at the Gate:*  Arranges transport, attends appointments.  *A* *Shared Understanding:*  Inquisitive, shows support and empathy, affect-focused, identifies main goals.  *The Shared Action Plan:*  Liaises with services. | *Trust & Engagement:*  Believes the practitioner is concerned for him.  Feels the practitioner demonstrates trustworthiness when they offer practical help.  Feels cared for.  Professional/ recipient power dynamic reduced.  *Mentalisation:*  Increased capacity to regulate emotions [short term; unsustained].  Mental health is supported.  *Supporting & Enabling:*  Engager fills gaps in service delivery.  Feels less frustrated in meetings [short term; unsustained].  *Increased autonomy:*  n/a | *Short term:*  Turns up to sessions [unsustained].  Engages in difficult decisions around their feelings.  Feels safe to disclose sensitive events/ experiences.  Increased understanding of the links between thoughts, feelings and actions.  *Medium term:*  Less likely to self medicate [unsustained].  Increases engagement with other services [unsustained].  *Long term:*  n/a  **No. of pts with improved CORE-OM score at 6 month follow up: 2** |
| **Group 4** | *Trust & Engagement:*  Limited trust established due to insufficient prison time to develop rapport, miscommunication resulting in the participant feeling ‘misled’, or failure to establish/ maintain contact with participants post-release. Engagement (if any) tended to occur primarily in prison, prior to participants’ release.  *Meeting at the Gate:*  Four of the six participants received release day support, three were met at the prison gate and one was met at probation. One declined the offer of support and the other was not met due to Engager practitioner absence.  *A Shared Understanding:*  No evidence of a shared understanding. Participants were appreciative of offers of practical support but appeared unwilling to commit to changing more ingrained patterns of behaviour.  *The Shared Action Plan:*  Some goals were identified pre-release but any steps to further these were stalled by early disengagement. Liaison with services tended to serve the primary purpose of attempting to re-engage participants post-release. | *Trust & Engagement:*  Quick wins, non-judgemental, shows concern, honest, actively listens.  *Meeting at the Gate:*  Attends appointments.  *A* *Shared Understanding:*  Affect-focused, inquisitive.  *The Shared Action Plan:*  Identifies resources to support goals, prioritises goals on individual priorities, develops achievable steps to attain goals, makes referrals, liaises with family and friends, liaises with service, plans out the last few weeks of support i.e. phase out contact, ensure support for the participant’s goals in place before ending, develop the participant’s individual and self-help skills. | *Trust & Engagement:*  Believes the practitioner is concerned for him.  Feels the practitioner demonstrates trustworthiness when they offer practical help.  Feels cared for.  Professional/ recipient power dynamic reduced.  *Mentalisation:*  Increased capacity to regulate emotions [short term; unsustained].  Mental health is supported.  *Supporting & Enabling:*  Engager fills gaps in service delivery.  Feels less frustrated in meetings [short term; unsustained].  *Increased autonomy:*  n/a | *Short term:*  Turns up to sessions [unsustained].  Feels supported to attend appointments [unsustained].  *Medium term:*  n/a  *Long term:*  n/a  **No. of pts with improved CORE-OM score at 6 month follow up: 2** |
| **Group 5** | *Trust & Engagement:*  Some trust established pre-release through offers of practical support and release day assistance. Trust lacked emotional depth as participants disengaged soon after release.  *Meeting at the Gate:*  Three of the five participants were met at the gate on release day and provided with assistance. The other two declined the offer.  *A Shared Understanding:*  No evidence of a shared understanding. Participants were appreciative of offers of practical support but appeared unwilling to commit to changing more ingrained patterns of behaviour.  *The Shared Action Plan:*  Some goals were identified pre-release but any steps to further these were stalled by early disengagement. Liaison with services tended to serve the primary purpose of attempting to re-engage participants post-release. | *Trust & Engagement:*  Quick wins, practical support, non-judgemental, shows concern, honest, actively listens.  *Meeting at the Gate:*  Attends appointments.  *A* *Shared Understanding:*  Affect-focused, inquisitive, identifies main goals.  *The Shared Action Plan:*  Identifies resources to support goals, liaises with service. | [Limited sources of data to make judgement] | [Limited sources of data to make judgement]  **No 6 month follow up CORE-OM scores collected.** |
