# Supplementary material for: Interrogating intervention delivery and participants’ emotional states to improve engagement and implementation: A realist informed multiple case study evaluation of Engager
Source: PLoS One. 2022 Jul 14;17(7):e0270691. doi: 10.1371/journal.pone.0270691 (PMC9282559; doi:10.1371/journal.pone.0270691)
Supplement: S4 File — (DOCX) [file pone.0270691.s004.docx]

S4 File: Patterns in reasoning behind sustained and non-sustained engagement: Exemplar Internal State Cases

*Crises but Coping:* Michael had a substance misuse problem, few support figures, and had spent much of his young adult life in and out of prison; spending short periods of time ‘clean’ in the community before resorting back to using drugs and committing further offences. He was mistrustful of services and didn’t readily engage with his Engager Practitioner to begin with, declining the offer of release day support several times before eventually changing his mind. Afterwards, the participant attributed the gate release support as being critical to his sobriety that day. Michael engaged consistently with the intervention for the first three months after his release, meeting up for at least one hour a week. Session content covered events, goals and action points, mood and emotions, substance misuse, and potential risks to self and others. Over time, Michael grew to perceive the Engager practitioner as someone he could be truly ‘open and honest’ with. He appreciated various aspects of the support, including having a mediatory presence at probation appointments (with whom Michael had a difficult relationship), exploring the motivations behind his substance use, and encouraging him to talk about his feelings instead of ‘bottling them up’ and ‘pushing them to one side’. Michael had a tendency to deflect blame for his actions onto others, and the rapport between him and his Engager practitioner was such that they explored this notion of responsibility. Michael experienced a severe relapse a few months after the intervention finished but instead of resorting to crime to fund the habit as he had done in the past, he independently sought help from a substance misuse service to get him sober again and acknowledged his own role in the crises of his life. This was the first time he had made such a choice and contributed to us characterising his state as ‘crises but coping’.

*Crises and Chaos:* James was a man in his 40s, who had spent most of his adult life in insecure residency, self-medicating on alcohol and other psychostimulant drugs, and had several convictions for minor offences related to his substance misuse. The Engager practitioner helped to secure him a hostel placement on release and initially he maintained engagement with the practitioner, attempting to make use of service support offers. However following a relapse to drugs he was evicted from the hostel and this led to a downward spiral of street homelessness and increasing dependence on illicit substances. The hostel eviction coincided with a public holiday whereby service support was operating at a reduced function and James wasn’t seen his by practitioner for a couple of weeks. When normal service resumed he was street-homeless and heavily self-medicating so keeping scheduled appointments and undertaking therapeutic work became very difficult given his near-constant inebriated state. Eventually James committed a further crime to fund his drug habit and he returned to prison. “I thought that there was not a lot of difference that you lot could make. Every day I was getting suicidal thoughts. Every pay day I was thinking about buying lots of heroin and ODing. Cos I didn’t see no end to it. It’s all going to hell. If I hadn’t have Od’d I would have purposely done it myself anyway”.

*Resigned Acceptance:* Lewis was apprehensive about his release from prison. He was not allowed contact with the mother of his children, had ongoing substance misuse issues, and was due to be released without accommodation. Pre-release he was interested in the possibility of getting a job and eluded to trying to stay clean post-release. However, despite offers of support from various agencies including potential temporary housing, gate release pick-up, possible job opportunities, and mental health support; he preceded to decline each attempt at engagement, eventually disengaging completely, deeming the efforts to change his life trajectory futile. “I go back and forth from mates’ houses [to sleep]. To be honest, that’s not too good but you know. I’m coping, I’m managing sort of. It’s stressful sometimes but other than that, it’s alright. I’ve always got somewhere to get my head down, I’m getting by”.

*Honeymoon:* Before his release, Matthew was confident that he would be able to get a job, support his pregnant girlfriend, avoid crime, and manage his substance use: ”I can’t exactly say that I’m never gonna drink again… I’m gonna wanna have a beer or whatever and it’s just all about controlling the substances instead of them controlling me”. Soon after his release Matthew found employment and stopped communicating with his Engager practitioner with assurances that he was well. However within 8 months of his release he had returned to heavy alcohol use and had attempted suicide: “when I come out last time I got back on the drink and my life just went downhill again”.

*Wilful Withdrawal:* Andrew was serving his first prison sentence for drug dealing (following several sentences at young offender institutions) and he perceived contact with services to be a ‘tick-box exercise’ to facilitate his release. Andrew was homeless but was unperturbed by this, and was intent on returning to his old lifestyle.

Before release he acknowledged that it was unlikely he would meet up with his practitioner in the community because he wanted to return to his previous lifestyle, and within months of his initial release Andrew was back in prison on a new charge: “I saw [the practitioner] once in town but that was unplanned, I just happened to see them. I didn’t want to get in touch with them, I didn’t want the help, I stopped listening. I wanted to carry on working and [the practitioner] offered to help me get work but I didn’t want the support. I don’t really know what Engager is”.
